# Management of Giant Thyroid Tumors in Patients with Multiple Comorbidities in a Tertiary Head and Neck Surgery Center

**DOI:** 10.3390/biomedicines12102204

**Published:** 2024-09-27

**Authors:** Daniela Vrinceanu, Mihai Dumitru, Andreea Marinescu, Crenguta Serboiu, Gabriela Musat, Mihai Radulescu, Matei Popa-Cherecheanu, Catalina Ciornei, Felicia Manole

**Affiliations:** 1 ENT Department, Faculty of Medicine, Carol Davila University of Medicine and Pharmacy, 020021 Bucharest, Romania; vrinceanudana@yahoo.com; 2 Imaging Department, Carol Davila University of Medicine and Pharmacy, 020021 Bucharest, Romania; andreea_marinescu2003@yahoo.com; 3 Molecular Biology and Histology Department, Carol Davila University of Medicine and Pharmacy, 050474 Bucharest, Romania; crengutas@yahoo.com; 4 ENT Department, Faculty of Dentistry, Carol Davila University of Medicine and Pharmacy, 020021 Bucharest, Romania; gabimusat@yahoo.com; 5 Thoracic Surgery Department, Carol Davila University of Medicine and Pharmacy, 020021 Bucharest, Romania; iulianmihai@yahoo.com; 6 Vascular Surgery Department, Carol Davila University of Medicine and Pharmacy, 020021 Bucharest, Romania; matei.cherecheanu@gmail.com; 7 Physiology Department, Carol Davila University of Medicine and Pharmacy, 020021 Bucharest, Romania; catalina.ciornei@umfcd.ro; 8 ENT Department, Faculty of Medicine, University of Oradea, 410073 Oradea, Romania; manole.felicia@gmail.com

**Keywords:** giant, thyroid tumors, comorbidities, multidisciplinary, challenges

## Abstract

**Background/Objectives**: The thyroid gland can represent the seat of development for giant tumors exceeding 10 cm in diameter. A retrospective analysis on 21 cases of giant thyroid tumors with comorbidities, operated in the ENT Department of the Bucharest University Emergency Hospital has been conducted. **Methods**: Giant benign tumors accounted for 28.57% of the cases studied and reached gigantic volumes in an average time interval of 3 years, with an average resection piece weight of 318 g. Malignant tumors accounted for 71.43%, with an average duration of evolution of 7 years, with an average resection piece weight of 581 g. **Results**: Dysphagia was present in all patients, dyspnea in 47.61% and dysphonia in 38.09% of cases. Total thyroidectomy is the gold standard in giant thyroid tumors, associated with radical neck dissection, extended to groups VI and VII in malignant tumors. In 10 of 11 cases of giant malignant thyroid tumors (90.90%), without anaplastic thyroid carcinoma, the survival at 5 years after multimodal oncologic treatment was favorable. Anticoagulant treatment increased the risk of postoperative reversible recurrent laryngeal nerve lesion. **Conclusions**: The management of giant thyroid tumors in patients with multiple comorbidities needs a multidisciplinary team including endocrinologist, radiologist, anesthesiologist, pathologist, ENT surgeon, thoracic surgeon, oncologist and radiotherapist.

## 1. Introduction

The thyroid gland, with its well-known butterfly shape, which typically weighs 30–50 g, can represent the seat of development for tumors that can reach impressive sizes. Usually, a cervical tumor can be categorized as giant or bulky if its diameter exceeds 10 cm [1].

Thyroid cancer is the most common endocrine malignancy, accounting for ~2.1% of all cancer diagnoses worldwide, with ~77% of these diagnoses occurring in women. Approximately 90% of all thyroid cancers are differentiated, meaning that they arise from thyroid follicular cells and are generally iodine-avid (able to “take up” iodine). Papillary thyroid carcinoma (PTC) is the most common histological type of differentiated thyroid cancer, followed by follicular thyroid carcinoma.

In the histological classification of thyroid gland tumors, epithelial tumors are the most numerous [2]. 

Among malignant epithelial tumors, the most common are papillary carcinoma (80–85%) and follicular carcinoma (15–18%). Among the rare forms of malignant epithelial tumors encountered are Hurthle cell carcinoma (3–5%), medullary carcinoma (3%) and undifferentiated, anaplastic carcinoma (1–2%) [3]. Thyroid tumor pathology is often interdisciplinary, being addressed equally by several specialties: endocrinology, the endocrinologist surgeon, the head and neck surgeon, the thoracic surgeon or even by the general surgeon. Diagnostic principles in thyroid gland tumors include endocrinological examination with thyroid hormone dosing in order to identify and preoperatively treat hyperthyroidism [4].

Thyroid and cervical ultrasound is a preoperative imaging exploration, often sufficient for simple poly nodular goiters, but which must be completed in the case of thyroid tumors, especially in large ones, with high-performance imaging. Fine needle aspiration (FNA) or ultrasound-guided core-biopsy is part of the routine preoperative assessment in a poly nodular goiter [5].

ENT clinical examination by fiberoptic laryngoscopy is necessary to highlight any recurrent paresis/paralysis. Thyroid scintigraphy provides information regarding cold nodules with high suspicion of malignant nodule, but it has limited use in the case of giant tumors. Imaging required for a giant thyroid tumor should be performed by a cervical CT with contrast or cervical MRI [6].

Regarding the management of malignant tumors of the thyroid gland, a multidisciplinary approach is required. The endocrinologist compensates for any preoperative hyperthyroidism, determines the optimal time of the surgical intervention, as well as the postoperative replacement treatment. He also monitors the patient clinically, sonographically and hormonally through TSH levels, in the postoperative period [7].

Surgical management of thyroid malignancies consists of total thyroidectomy with preservation of the recurrent laryngeal nerves and at least one parathyroid gland associated with radical neck dissection (RND) that must also include cervical ganglion groups V, VI and VII. There are studies that show that unilateral thyroid lobectomy in certain forms of thyroid nodules may be equivalent in terms of results and long-term prognosis, but this statement remains valid only for small nodules. In voluminous tumors with extracapsular extension, salvage surgery is performed with total thyroidectomy with neck dissection and ablation also of invaded adjacent structures like infrahyoid muscles. Surgery is correlated with TNM classification [8].

The pathologist has an essential input in the treatment team by formulating a preliminary diagnosis after FNA, but especially by formulating a diagnosis on frozen sections. Paraffin histopathological examination and immunohistochemical examination formulate a diagnosis of certainty and can certify the shift of a papillary carcinoma with a long evolution to an anaplastic carcinoma [9].

The radiologist provides the necessary acquisitions for a correct and complete preoperative assessment, providing essential details regarding relationships with the great cervical vessels, possible internal jugular vein thrombosis, mediastinal extension, or invasion of cavitary organs, such as the larynx, pharynx, esophagus. The cardiologist contributes by preoperative cardiac compensation. In the multimodal treatment of malignant thyroid tumors, postoperative radiotherapy intervenes. In differentiated (follicular or papillary) carcinomas, radioactive iodine therapy is useful, while in undifferentiated forms, external beam radiation therapy is applied, as in all head and neck cancers. The oncologist proves its usefulness by combining chemotherapy in forms of anaplastic carcinoma [10].

Data from Italy reveal that cardiovascular pathology seems higher as incidences in cases with enlarged thyroid nodules [11]. Metabolic syndrome is an increased public health problem as tends to be associated with a higher risk of developing aggressive thyroid carcinoma [12]. Older age is now increasing in the possible indications for preferring percutaneous radiofrequency ablation of thyroid carcinomas in cases ineligible for surgery [13].

All previously mentioned types of malignant tumors can develop as giant thyroid tumors that impose numerous challenges in surgical management.

In the present article, we aimed to present our experience in managing these challenges in the management of giant thyroid tumors in a tertiary head and neck surgery center within a multidisciplinary hospital.

## 2. Materials and Methods

We performed a retrospective analysis on 21 cases of giant thyroid tumors with comorbidities, operated in the ENT Department of the Bucharest University Emergency Hospital, between January 2012 and December 2021. Our department is a reference center for head and neck oncological surgery. Patients admitted to the study were referred to our department due to experience in oncological cervical surgery, as well as due to the presence of at least one comorbidity, with the need for monitoring and treatment in a multidisciplinary hospital. Inclusion criteria were patients with bulky thyroid tumors (more than 10 cm in craniocaudal dimension), located in at least one thyroid lobe and having at least one major comorbidity such as cardiovascular disease, vascular neurological disease, degenerative neurological disease and chronic lung disease. Patients with thyroid tumors smaller than 10 cm in cranio-caudal diameter, patients without comorbidities and patients with a histopathological result of lymphoma type were excluded. Patients were referred to our department, which is a tertiary head and neck surgery center in a multidisciplinary hospital, by other endocrinological surgery centers where they could not be approached surgically due to anesthetic and surgical risks, as well as of the impossibility of a multidisciplinary approach to cases. These are the aspects that increase the difficulty of such complex cases, because the patients scarcely controlled their associated pathology. Unfortunately, apparently controllable associated pathology presents an increased burden to the patient with giant thyroid tumors. The preoperative protocol consisted of endocrinological examination and full thyroid hormone profile dosage (TSH, free T3, free T4, calcitonin, parathormone, thyroglobulin); ENT clinical examination, with nasopharyngolaryngeal flexible endoscopy; thyroid ultrasound, cervical and thoracic CT with iv contrast agent; in cases that represented hyperthyroidism, cervical and thoracic CT examination was replaced by cervical and thoracic MRI examination; in one case, bilateral carotid and subclavian angiography was performed, with preoperative selective embolization; cardiological examination; preanesthetic exam. Patients who presented hyperthyroidism were appropriately treated and compensated preoperatively by the endocrinologist. All patients underwent total thyroidectomy with functional or radical neck dissection. All patients underwent extemporaneous intraoperative examination, with sections on ice, then histopathological examination with paraffin, with immunohistochemistry. All operated patients were admitted to the ATI postoperatively for a minimum of 48 h and received replacement therapy with synthetic thyroid hormones Euthyrox 100 micrograms/day and calcium preparations with vitamin D3 p.o., starting 48 h postoperatively. In the retrospective analysis, patients with a histopathological result of follicular adenoma, papillary carcinoma, follicular carcinoma, medullary carcinoma, or anaplastic carcinoma were included.

The long period of time of almost 10 years implied that some aspects from the records, such as all daily laboratory results, all daily measurements of temperature or blood pressure, were unavailable in some of the records. Therefore, we focused only on the data that were consistently available for all the cases included in the study.

## 3. Results

From the perspective of distribution by gender, there was a higher percentage of men than women, with practically 61.90% of the cases being represented by male patients. 

**Figure 1:**
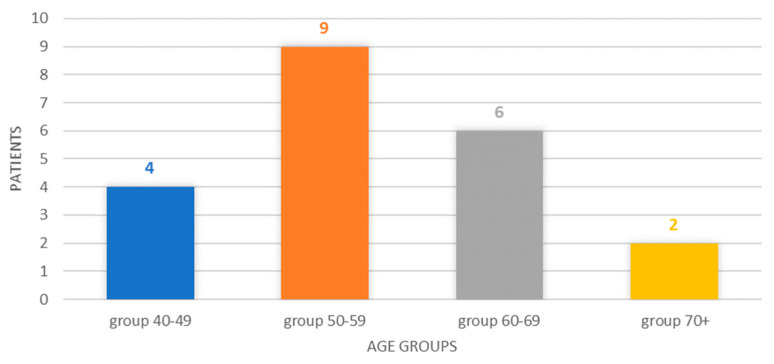
Chart showing the distribution of cases by age group.

The breakdown by age group reveals a classification of four cases in the 40–49 age group (19.05%), six cases corresponding to the 50–59 age group, six cases in the 60–69 age group (28.57%), respectively, and two cases in patients older than or at least 70 years old (9.52%). The 50–59 age group represented 42.85% of the cases, and the 40–49 and 50–59 age groups represented, cumulatively, 61.90% of the cases, Figure 1.

Distribution according to the histopathological result confirmed by immunohistochemistry the following: giant follicular cyst on poly nodular goiter, with intracystic hemorrhage—one case; follicular adenoma on poly nodular goiter—five cases; papillary carcinoma—seven cases; follicular carcinoma—two cases; medullary carcinoma—two cases; anaplastic carcinoma—four cases. Rare forms of thyroid cancer represented eight cases in our group, while papillary carcinoma was present in seven cases. It is noteworthy that the patients with benign thyroid tumor pathology accounted for 28.57%. On the other hand, there were four patients with anaplastic carcinoma, representing 19.04% of the studied cases.

**Figure 2:**
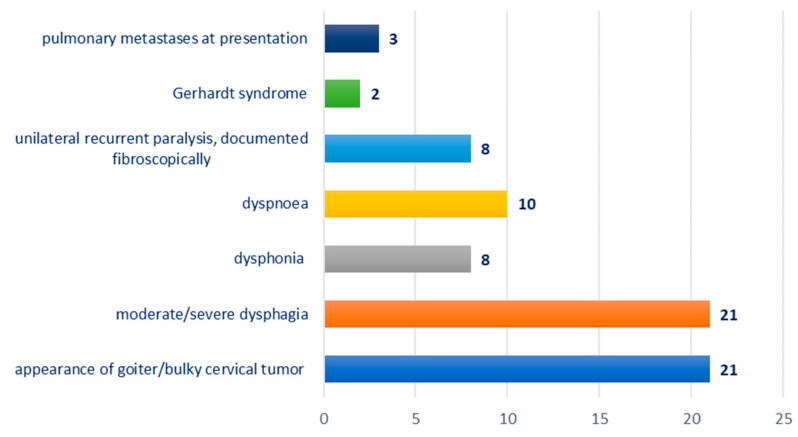
Chart depicting the distribution of cases according to clinical presentation.

Distribution according to the clinical picture at presentation: appearance of goiter/bulky cervical tumor—21 cases; moderate/severe dysphagia—21 cases; dysphonia—eight cases; dyspnea—10 cases; unilateral recurrent paralysis, documented fibroscopically—eight cases; Gerhardt syndrome—two cases; pulmonary metastases at presentation—three cases; Figure 2.

**Figure 3:**
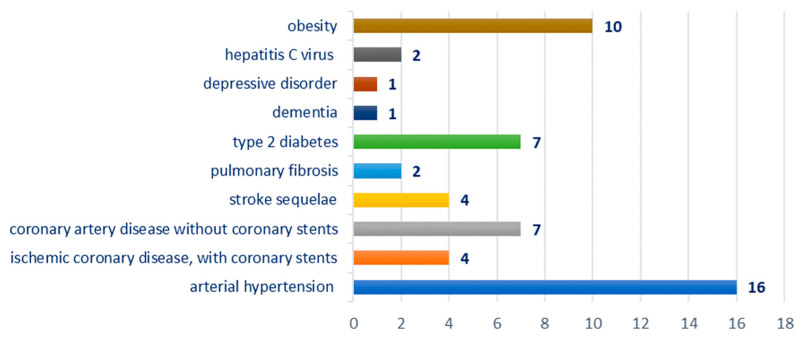
Chart showing the distribution of cases according to the associated comorbidity.

Breakdown by type of comorbidities: hypertension—16 cases; ischemic coronary disease—11 cases, of which four cases were with coronary stents; stroke sequelae—four cases; pulmonary fibrosis—two cases; obesity—10 cases, type 2 diabetes—seven cases; hepatitis C virus—two cases; dementia—one case; depressive disorder—one case; Figure 3.

Distribution according to the type of surgical interventions performed: total left thyroid lobectomy—one case; total thyroidectomy—five cases; total thyroidectomy, with the infrahyoid muscle resection—three cases; total thyroidectomy + neck dissection (radical/functional)—12 cases, of which three cases were with radical neck dissection and nine cases were with functional neck dissection; sacrifice of recurrent laryngeal nerve without reconstruction—two cases with unilateral lesion; tracheostomy—two cases; gastrostomy—one case.

Distribution by type of multidisciplinary team of surgeons: head and neck surgeon—21 cases; thoracic surgeon—four cases; vascular surgeon—one case.

Distribution by type of orotracheal intubation: on videolaryngoscope—19 cases; awake intubation on the bronchoscope—two cases.

**Figure 4:**
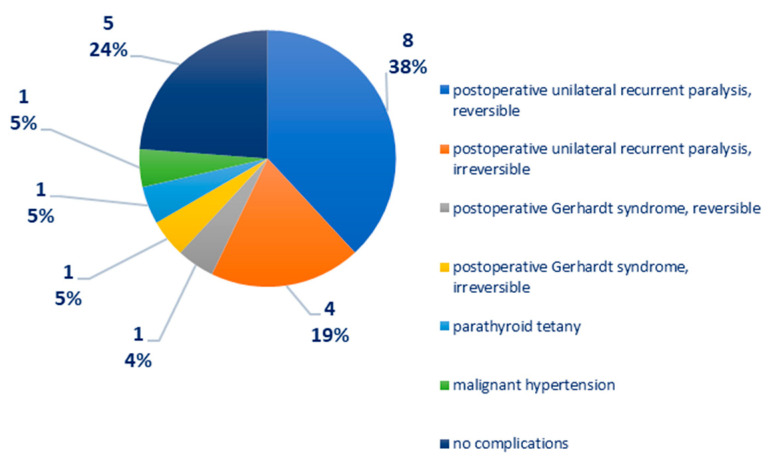
Chart depicting the distribution of cases according to postoperative complications.

Distribution according to postoperative complications: recurrent unilateral postoperative paralysis—12 cases, of which eight cases were reversible, four cases were irreversible; postoperative Gerhardt syndrome—two cases, of which one case is reversible, one case is irreversible; parathyroid tetany—one case; malignant arterial hypertension—one case, Figure 4. In one case of medullary thyroid carcinoma, there was a right phrenic nerve paresis and a paralytic ileus, reversible after 72 h of cortisone treatment.

Distribution of cases according to adjuvant treatment: endocrinological substitution treatment, under the supervision of the endocrinologist—21 cases; radioactive iodine—nine cases; external radiotherapy—two cases; chemotherapy—three cases; immunotherapy—two cases.

**Figure 5:**
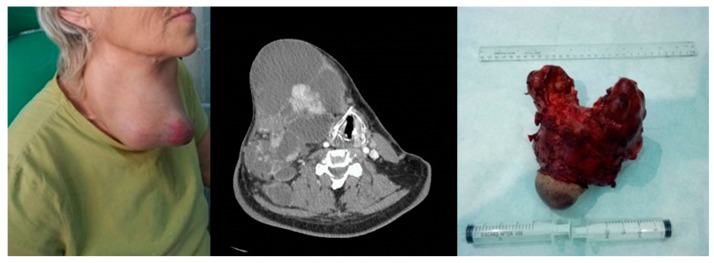
Anaplastic thyroid carcinoma. Left image—clinical aspect. Center image—axial CT scan with tumor displacement of the airway. Right image—resection piece 750 g.

Breakdown by evolution: the LTS giant follicular cyst case was followed at 1 month and 3 months and then endocrinologically discharged, and the five poly nodular goiter follicular adenoma cases were followed at 1 month and 6 months and entered in the endocrinological dispensary. Among the 21 cases of the studied group, there were 16 survivors at 5 years, in oncological monitoring at 3 months. The four cases of anaplastic carcinoma resulted in death, as well as one case of papillary carcinoma with multiple metastatic disease. The outcome was through tracheal invasion—four cases, respectively, through multiple metastatic disease—one case. Among the four cases of anaplastic carcinoma, three cases followed chemotherapy, with resumption of evolution and exitus, respectively, and one case had a resumption of evolution 6 weeks postoperatively, with exitus, Figure 5.

## 4. Discussions

In the studied group, male patients were respondents, representing almost 2/3 (61.90%) of patients with bulky thyroid tumors and multiple comorbidities. The mean age of patients at presentation was 58 years, with an age range between 44 and 77 years. The most numerous were patients in the 50–59 age group, representing 42.85% of the cases, followed by the 60–69 age group, representing 28.57% of the cases. Basically, 61.90% of patients with giant thyroid tumors were between the ages of 40 and 59, representing younger ages than those of 60–69 and 70 and over, which accounted for 28.57% and, respectively, 9.52% of cases. The occurrence of giant thyroid tumors is correlated, rather, with a younger age. The duration of the evolution in time, until the first presentation to the doctor, had a median of 6 years, with a range between 3 months and 11 years. These aspects are in concordance with a similar study from Korea where 73% of the patients were between 40 and 69 years of age [14]. A history of autoimmune thyroid disease poses specific problems for patients in the 35–55 age group according to a study on 237 patients with Graves Disease with Synchronous Thyroid Nodules [15]. A study from Lithuania showed that a total of 5664 cases of thyroid cancer were registered in the entire Lithuanian population in the last 25 years; 817 cases in the age group from 0 to 19 years at the time of the Chernobyl accident, and 266 cases in the age group from 0 to 9 years, due to the exposure to the Chernobyl accident [16]. In China, age-standardized mortality rates decreased in specific age groups and genders, especially among women. Conversely, the age-standardized mortality rate significantly increased in groups aged over 70. The mortality/incidence ratio exhibited a declining trend, but this decrease was less noticeable in men and the group aged over 70 [17]. Thus, our findings show that the increased trend of a higher incidence of giant carcinoma of the thyroid gland is part of the general increased trend of general carcinoma of the thyroid gland incidence.

The clinical picture at presentation included a bulky cervical tumor, with skin invasion in three of the cases. It should be noted that the reason for presenting to the doctor was not the unaesthetic appearance of the tumor, but the appearance of another clinical sign such as dysphagia, dysphonia or dyspnea. Dysphagia was present in all cases and was objectively evaluated by pharyngoesophageal barite transit and functional endoscopic evaluation of swallowing (FEES). From a subjective point of view, swallowing disorders were subjectively assessed by the patients by completing an EAT-type questionnaire. This is an aspect also reported in other cases with a long-neglected evolution leading to extreme weight loss at the moment of admission to the hospital [18].

Dysphonia was present in eight cases (38.09%) and dyspnea in 10 cases (47.61%), so practically in half of the cases. In the six cases with benign tumor pathology, the clinical symptoms were limited to the presence of the thyroid tumor and dysphagia, except for the case of giant follicular cyst which also associated dysphonia and dyspnea and cervical pain, possibly also in a post-traumatic context (two episodes of syncope). The syncope can be generated through a direct compression mechanism on the major blood vessels of the neck as shown by other cases also reported [19].

Unilateral recurrent paralysis was present in eight cases, and Gerhardt’s syndrome in two cases, including one female patient, 53 years old, with papillary carcinoma and one case of a female patient, 57 years old, with anaplastic carcinoma. There was no case of recurrent paralysis at presentation in patients with bulky benign thyroid tumors. The presence of this neurological disorder at admission is associated with a malignant histological diagnosis. Pulmonary metastases at presentation in three cases were associated with longer evolution and the large volume of the primary tumor. Sometimes, the involvement of the recurrent laryngeal nerve can be both in the intrathoracic course through goiter-like pressure and in the neck through sheer tumor mass displacement [20].

The comorbidities of the patients in the studied group fall into vascular and cerebrovascular diseases (hypertension, ischemic coronary disease with/without coronary stents, stroke sequelae, type 2 diabetes), neurological and psychiatric conditions (dementia, depressive disorder). A special remark deserves to be made on patients with antiplatelet and/or anticoagulant treatment who assume a risk of postoperative complications; therefore, the resumption of anticoagulant therapy with low molecular weight heparin was conducted 16 h postoperatively. Metabolic syndrome was reported as a negative predictor for the evolution of thyroid carcinoma [21].

A study on 21,509 patients with a median age of 72 years (range 66–106), revealed that 4168 (19.4%) died of other causes and 2644 (12.3%) died of thyroid cancer during the study period. For differentiated thyroid cancer patients, the likelihood of dying from other causes exceeds the likelihood of dying from thyroid cancer, whereas the opposite is true for anaplastic thyroid cancer. For medullary thyroid cancer, after 6.25 years, patients are more likely to die from other etiologies than thyroid cancer [22]. In a bigger cohort, after a median follow-up of 101 months, 23,040 (13.3%) deaths occurred, of which 29.1% and 21.7% were attributable to thyroid cancer and cardiovascular disease (CVD), respectively [23]. We cannot generalize our findings, but they are concurring with the findings in the general population of thyroid carcinoma patients. The added impact of the dimensions of the tumor, local impact on blood vessels and the airway increases the cardiovascular risk of the patients with giant thyroid carcinoma.

From the point of view of surgical interventions performed in giant thyroid tumors, total thyroidectomy is certainly the gold standard. One case of giant follicular cyst of the left thyroid lobe received total left thyroid lobectomy, in order not to expose the patient to additional risks, being a 77-year-old patient with dementia, who clinically presented dysphagia, dyspnea and dysphonia. In all surgical interventions, an extemporaneous examination was used with sections on ice, which facilitated our operative decision. Radical neck dissection included lymph node groups VI and VII in all cases with malignant extemporaneous examination. In four cases, a thoracic surgeon joined the operative team, to ensure the mediastinal dissection, and in one case the aid of a vascular surgeon in the team was solicited to ensure the dissection of the thoracic duct in the case of a patient with anaplastic carcinoma developed predominantly on the left side. The tumor-invaded recurrent laryngeal nerve was resected without reconstruction in two cases (unilateral lesion) [24].

There are still debates regarding the concept of extended neck dissection in cases with giant thyroid tumors. The cases in the study group tend to some of the characteristics of an extended neck dissection which refers to the excision of one or more additional lymph node clusters or nonlymphatic entities, or both, that are not covered by the radical neck dissection (examples of these lymph node clusters include parapharyngeal, superior mediastinal, paratracheal; examples of nonlymphatic entities include the recurrent laryngeal nerve and paraspinal muscles) [25]. Salvage surgery has a high risk of complications and it may be worthwhile when macroscopic curative resection is available. The decision should be made considering various factors including curability, risk of surgical procedure, functional outcome and life expectancy [26].

The surgical interventions were, except for cases with benign tumor pathology, of long duration, on average 428 min (over 7 h). The average bleeding in these procedures for giant malignant thyroid tumors was 335 mL and required intraoperative transfusion of labile blood products (erythrocyte mass, fresh frozen plasma). A case of voluminous anaplastic carcinoma received preoperative angiography with selective embolization of the superior thyroid artery and thyrocervical trunk, without operatively finding a significant change in bleeding, despite the embolization (in that case, with an intraoperative bleeding of approximately 500 mL). Large populational studies credit these types of tumors with only a 1.3% risk of bleeding [27].

It should be noted that postoperative complications recorded 12 cases of recurrent unilateral paralysis, of which eight were reversible and four irreversible, as well as two postoperative Gerhardt syndromes, of which one was reversible and one was irreversible. It is worth noting that two cases of reversible recurrent paralysis and one case of reversible Gerhardt syndrome were found in patients with benign tumors (three of six cases). In these cases of benign tumor pathology, the recurrent paralysis is explained by the large tumor volume that compresses the nerve that involves laborious dissection, and especially by the vascular status of the patient and the use of antiaggregant, oral anticoagulants that can cause microhemorrhages in the nerve sheath, which can be associated with a postoperative transient recurrent paralysis. One case developed postoperative Gerhardt syndrome in an obese patient with a history of acute myocardial infarction and coronary stents, who was being treated with oral anticoagulants. The occurrence of postoperative Gerhardt syndrome, after extubating, required the maintenance of intubation for another 48 h and treatment with systemic corticosteroids + group B vitamins, and later tracheotomy on the same thyroidectomy incision. Fortunately, the evolution was favorable, and the patient was able to be decannulated after 2 months. It is useful in preventing postoperative recurrent paralysis to avoid dissection and the use of monopolar electrocautery medial to the recurrent line without prior identification of the nerve [28].

Differentiated thyroid carcinoma has a promising prognosis if detected in early stages contrary to prognosis of the pathology subtypes included in the present group of giant thyroid tumors. The overall survival for patients with advanced or metastatic differentiated thyroid carcinoma resistant to radioactive iodine therapy is poor, with 5- and 10-years survival rates of 50% and 10%, respectively [29]. In a study on almost 300 cases of papillary thyroid carcinoma, recurrence occurred in 20 cases (7.3%) and lympho-vascular invasion and lateral cervical lymph node metastasis made significant independent contributions [30]. The major indicator in a low quality of life for the patients undergoing thyroid surgery is the necessity of tracheostomy. A study from Japan concluded that super-radical procedures may be indicated in select patients to avoid mortality due to locally uncontrolled disease at the expense of tracheostomy [31]. Our study focusing on giant thyroid tumors underlines that extensive surgeries are associated with a reserved prognosis, diminished survival rates and poor quality of life implied by salvage surgery procedures.

There was a single case of parathyroid tetany in a patient with anaplastic carcinoma. In these giant tumor cases, the preoperative identification of the parathyroid glands is very difficult [32].

Regarding the distant evolution, 16 (76.19%) of the 21 cases of giant thyroid tumors in patients with multiple comorbidities survived, following a program of oncological monitoring every 3 months. All cases of anaplastic carcinoma resulted in death (four of four) and one case of papillary carcinoma with multiple metastatic disease, confirming data from the literature regarding this type of neoplasia [33].

The molecular biology of thyroid carcinoma is very complex ranging from gene mutations, gene amplifications and copy-number gains, gene translocations, aberrant gene methylation, to altered signaling pathways and progressive molecular alterations [34]. Anaplastic thyroid carcinoma presents various subtypes: Type 1 *BRAF*-positive, with a genetic landscape similar to papillary thyroid carcinoma; Type 2 *NRAS*-positive which may originate from follicular thyroid carcinoma; Type 3 which carries *RAS* mutations or more atypical ones (e.g., *PTEN*, *NF1* and *RB1*) and is likely to originate from Hürthle cell carcinoma; and Type 4 mixed, which harbor loss-of-function genetic alterations and mutations in the genes of cell-cycle regulations (*CDKN2A* and *CDKN2B*) [35]. This leads to uncontrolled activity of various signaling pathways: the MAPK, PI3K–AKT, nuclear factor-κB (NF-κB), RASSF1–mammalian STE20-like protein kinase 1 (MST1)–forkhead box O3 (FOXO3), WNT–β-catenin, hypoxia-inducible factor 1α (HIF1α) and thyroid-stimulating hormone (TSH)–TSH receptor (TSHR) pathways [36].

For patients with locoregionally confined (stage IVA or IVB) anaplastic thyroid carcinoma, chemotherapy is applied as concurrent radiochemotherapy or adjuvant therapy. The drug candidates are taxanes, anthracyclines and platinum derivatives. Weekly regimens are recommended when administered concurrently with radiotherapy [37].

In summary, we consider the management of bulky thyroid tumors in patients with multiple comorbidities to be performed in a multidisciplinary team pre, intra and postoperatively. This multidisciplinary team necessarily includes the endocrinologist, the imager, the anesthetist, the anatomopathologist, the neck surgeon, sometimes the thoracic surgeon, the oncologist and the radiotherapist, which sometimes makes the timing difficult. The anesthetist’s challenges relate to difficult intubation requiring video laryngoscopy or awake bronchoscope intubation, intraoperative transfusion, postoperative intensive care and cardiac monitoring. Surgical challenges relate to the preservation of the recurrent laryngeal nerves, preservation of at least one parathyroid gland, preservation of the integrity of the pharyngoesophageal axis and control of hemostasis. The surgical team involved in the ablation of bulky tumors of the thyroid gland must be physically and psychologically trained, because it is a long surgery, with experience gained over time. Just as functional outcomes differ between an occasional thyroid surgeon and a routine thyroid surgeon, the challenges of surgical management in bulky tumors are better addressed in multidisciplinary teams trained in this type of pathology.

### Limitations

There are some limitations to the present study due to the fact that it is a transversal retrospective study design. The number of subjects in the study is limited because these types of tumors are rare. The complex team necessary to manage such difficult surgical cases is available only in few tertiary healthcare units. Future studies on a wider group of patients are still necessary for clearing the indication of salvage surgery in these advanced carcinoma cases.

## 5. Conclusions

The data presented in this study strengthen the hypothesis that the long evolution of a thyroid tumor is associated with malignancy. Total thyroidectomy is the gold standard in giant thyroid tumors, associated with neck dissection, extended to groups VI and VII in malignant tumors. Cardiological, neurological and endocrinological monitoring prevented major complications in the postoperative period. The presence of comorbidities did not negatively influence the postoperative evolution, but it was important to know them for investigation and specialized monitoring. The unfavorable prognosis was associated with the HP type of anaplastic carcinoma. Extending the research to larger study groups may help in generalizing the findings.

## Data Availability

All data are available from the corresponding author upon reasonable request due to the increased size of the dataset.
